# Use of Linear Free Energy Relationships (LFERs) to Elucidate the Mechanisms of Reaction of a γ-Methyl-β-alkynyl and an *ortho*-Substituted Aryl Chloroformate Ester

**DOI:** 10.3390/ijms13010665

**Published:** 2012-01-10

**Authors:** Malcolm J. D’Souza, Jaci A. Knapp, Gabriel A. Fernandez-Bueno, Dennis N. Kevill

**Affiliations:** 1 Department of Chemistry, Wesley College, 120 N. State Street, Dover, DE 19901, USA; E-Mails: jaci.knapp@email.wesley.edu (J.A.K.); gabriel.fernandez@email.wesley.edu (G.A.F.-B.); 2 Department of Chemistry and Biochemistry, Northern Illinois University, DeKalb, IL 60115, USA

**Keywords:** solvolysis, nucleophilicity, ionizing power, γ-Methyl-β-alkynyl chloroformate, 2-butyn-1-yl-chloroformate, aryl chloroformate, 2-methoxyphenyl chloroformate, Grunwald-Winstein equation, Linear Free Energy Relationships (LFERs)

## Abstract

The specific rates of solvolysis of 2-butyn-1-yl-chloroformate (**1**) and 2-methoxyphenyl chloroformate (**2**) are studied at 25.0 °C in a series of binary aqueousorganic mixtures. The rates of reaction obtained are then analyzed using the extended Grunwald-Winstein (G-W) equation and the results are compared to previously published G-W analyses for phenyl chloroformate (**3**), propargyl chloroformate (**4**), *p*-methoxyphenyl choroformate (**5**), and *p*-nitrophenyl chloroformate (**6**). For **1**, the results indicate that dual side-by-side addition-elimination and ionization pathways are occurring in some highly ionizing solvents due to the presence of the electron-donating γ-methyl group. For **2**, the analyses indicate that the dominant mechanism is a bimolecular one where the formation of a tetrahedral intermediate is rate-determining.

## 1. Introduction

**Figure 1:**
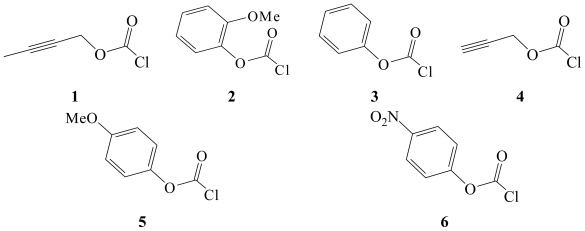
Molecular structures of 2-butyn-1-yl chloroformate (**1**), 2-methoxyphenyl chloroformate (**2**), phenyl chloroformate (**3**), propargyl chloroformate (**4**), 4-methoxyphenyl chloroformate (**5**), and *p*-nitrophenyl chloroformate (**6**) are shown where the C=O is *syn* with respect to the alkynyl or aryl moiety; *i.e.*, the halogen atom is in a *trans* position with respect to the alkynyl or aryl group.

γ-Methyl-β-alkynyl and mono substituted phenyl chloroformate esters such as 2-butyn-1-ylchloroformate (**1**) and 2-methoxyphenyl chloroformate (**2**) shown in Figure 1, have found use in the preparation of symmetrical urea’s that have patented herbicidal control applications [1,2] against certain weeds, fungi, and bacteria. With the recent introduction of novel synthetic methodology [3–5], the interest in the synthetic utility of such alkynyl and aryl esters is further enhanced due to their supplemental increased use in pharmaceutical formulations.

Linear free energy relationships (LFERs) [6–8] such as the Grunwald-Winstein (G-W) equations (Equations 1 and 2) [9,10] are often used to correlate [11,12] the specific rates of solvolysis of organic substrates to the solvent ionizing power [13–17] and solvent nucleophilicity values [18–20]. To commemorate the sixtieth anniversary of the original Grunwald-Winstein equation (Equation 1) [9] we documented [12] the utility of these G-W LFERs (Equations 1 and 2) in the analysis of a variety of organic compounds including a collection of chloroformate esters. Since this comprehensive review, several additional papers [21–43] have appeared; some corroborating the results obtained using Equations 1 and 2 [12] and proving that these LFERs are very successful in the elucidation of the solvolysis mechanisms for an assortment of acid chlorides.

(1) $$\text{log }(k/k_{o})={mY}_{\text{X}}+c$$

(2) $$\text{log }(k/k_{o})={lN}_{\text{T}}+{mY}_{\text{X}}+c$$

In the Grunwald-Winstein Equations 1 and 2, *k* and *k_o_* are the specific rates of solvolysis of a substrate in a given solvent and in the standard solvent (80% ethanol), respectively, *m* represents the sensitivity to changes in the solvent ionizing power *Y*_X_ (based on the solvolysis of 1- or 2-adamantyl derivatives) [13–17], *l* is the sensitivity to changes in solvent nucleophilicity *N*_T_ (based on the solvolysis of *S*-methyldibenzothiophenium ion) [18–20], and *c* is a constant (residual) term.

For substrates that ionize via anchimeric assistance (*k*Δ), or where conjugation allowed delocalization of an adjacent π-system, we proposed [44,45] adding an additional term, the aromatic ring parameter *I*, to Equations 1 and 2, to give Equations 3 and 4. In Equations 3 and 4, *h* represents the sensitivity of solvolyses to changes in the aromatic ring parameter *I*.

(3) $$\text{log }(k/k_{o})={mY}_{\text{X}}+hI+c$$

(4) $$\text{log }(k/k_{o})={lN}_{\text{T}}+{mY}_{\text{X}}+hI+c$$

**Scheme 1:**
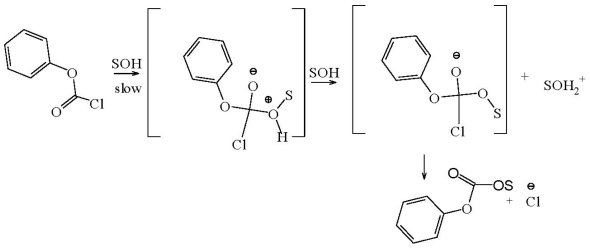
Stepwise addition-elimination mechanism through a tetrahedral intermediate for phenyl chloroformate (**3**).

The solvolysis of substituted phenyl chloroformates [11,12,22,24,46–55], including the effects of aminolysis [56,57] and micellar aggregates [58–60] on rates of reaction, has been extensively studied in a wide variety of solvents. A thorough multiple regression analysis employing Equation 2 using various subsets of solvolytic rate data for phenyl chloroformate (**3**) in 49 solvents of widely varying nucleophilicity and ionizing power values, resulted in the proposal [12,50,54] that **3** solvolyzes by an addition-elimination pathway with the addition step being rate-determining. Using Equation 2, the sensitivity values for **3** of 1.66 obtained for *l* and 0.56 obtained for *m*, are now taken [12,21,22–26, 28–39,41,42,50,54] as typical values that are to be expected for solvent attack at an sp^2^ hybridized carbonyl carbon and involving the rate-determining formation of a tetrahedral intermediate (Scheme 1).

Recently we showed [35] that the inductive ability of the alkynoxy group in propargyl chloroformate (**4**) when compared to that of the phenoxy group in phenyl chloroformate (**3**) is reduced, as the alkynyl group is pushed out of the plane of the ester oxygen due to the presence of the additional carbon between the alkyne and the ester oxygen in **4**. Additionally, we demonstrated [35] that **4** and **3** solvolyze by a similar bimolecular addition-elimination pathway with a rate-determining addition step in a variety of solvents including those with appreciable fluoroalcohol content. On the other hand, the former war gas isopropenyl chloroformate, was shown [37] to exhibit a superimposed unimolecular ionization (S_N_1) mechanism in the strongly hydrogen-bonding 1,1,1,3,3,3-hexafluoro-2-propanol (HFIP) and 97% 2,2,2-trifluoroethanol (TFE) mixtures with water.

It has been well documented [10,12,32,45,61,62] in LFER analyses such as when using Equations 2–4, that the inclusion of mixtures of fluoroalcohols is important to avoid multicollinearity effects when studying substrates like chloroformate esters [54,55]. We recently demonstrated [55] that the inclusion of additional highly ionizing 2,2,2-trifluoroethanol (TFE) and 1,1,1-3,3,3-hexafluoro-2-propanol (HFIP) mixtures decreased the *h* value of *p*-methoxyphenyl chloroformate (**5**) from 0.85 reported [54] for 31 solvents to 0.29 for 44 solvents [55]. The latter aromatic ring parameter number was also associated with a large probability (0.114) that the *h* term was statistically insignificant [55].

We previously compared [24] the Grunwald-Winstein (Equation 2) analyses obtained for **3** [12,50,54], **5** [54,55], and *p*-nitrophenyl chloroformate (**6**) [24] in 38 common solvents and determined *l* values of 1.59, 1.58, and 1.69, and *m* values of 0.54, 0.57, and 0.46 respectively. Other groups [11,48,53,63] have rightly stressed the importance of general-base catalysis occurring in the solvolyses of these three aryl chloroformates which was indicated by large observable kinetic solvent isotope effects (KSIEs) in methanol and methanol-d (*k*_MeOH_/*k*_MeOD_). In an identical set of solvents, the observed *l*/*m* ratios of 2.94 for **3**, 2.77 for **5**, and to 3.67 for **6** implies an earlier transition-state for **6** due to the presence of the strongest inductive interaction because of the *p*-nitro group and a decrease in the importance of general base catalysis in going from **6** to **3** to **5** [24].

Very recent studies of carbamoyl chlorides using Equation 2 proposed ionization reactions for 4-morpholinecarbonyl chloride (**7**) [64] and 1-piperidincarbonyl chloride [65]. A little over a year ago, in a personal communication to Koo [64,65] it was pointed out that he had overlooked our previous manuscript [66] published about 15 years earlier detailing the application of Equation 2 to the solvolyses of 4-morpholinecarbonyl chloride (**7**). Our work was at 25.0 °C [66], and the six more recently reported measurements at the same temperature [64] are in very good agreement. Also, the *l* and *m* values of 0.71 and 0.65 obtained for **7** are in excellent agreement with our earlier determined values of 0.74 and 0.66. The *l*/*m* ratio of 1.12 obtained [66], conforms to the expected range of the other carbamoyl esters [12] and implies a strong nucleophilic solvation effect at the developing carbamoyl carbocation [12,66]. The reason that the Koo group [64,65] continue to overlook our work is probably because we named compound **7** as 4-(chloroformyl)morpholine (from the Aldrich catalog); which is a synonym [67] for 4-morpholinecarbonyl chloride.

As shown in Figure 1, 2-butyn-1-yl chloroformate (**1**) and propargyl chloroformate (**4**) differ only due to the presence of an adjoining methyl group on the β-triple bond in **1**. In order to investigate whether the electron supplying effect of this γ-methyl group will have an additive influence on the solvolytic transition state, we have studied the solvolyses of **1** in eighteen binary organic-aqueous solvents with very different nucleophilic and ionizing abilities. Additionally in this article, we have analyzed the effect of the presence of a methoxy group in the 2-position in *o*-methoxyphenyl chloroformate (**2**) in seventeen solvents, and compared this data to those earlier obtained for the presence of a methoxy group in the 4 position in *p*-methoxyphenyl chloroformate (**5**) in identical solvents. To further analyze substituent effects, we have compared the solvolysis rates of **2** with the previously published compilation of rate data for phenyl chloroformate (**3**) and *p*-nitrophenyl chloroformate (**6**).

## 2. Results and Discussion

The experimental first-order specific rate constants that are listed in Table 1 were obtained for 2-butyn-1-yl chloroformate (**1**) in 18 binary aqueous-organic solvents of widely varying nucleophilicity and ionizing power values at 25.0 °C. Also listed are the previously published rate data for the sovolysis of propargyl chloroformate (**4**) [35] at 25.0 °C and the literature *N*_T_ [18–20] and *Y*_Cl_ [13–17] values.

From the data reported for substrate **1** in Table 1, one observes that the rates of reaction increase as the water content increases in the aqueous-organic mixtures and with the increase in ethanol (EtOH) content in the TFE-EtOH mixtures. This progression suggests that the solvents nucleophilic component plays an important role at developing transition state.

Also tabulated in Table 1 are the rate ratios (*k***_1_**/*k***_4_**) in the commonly studied solvents. In the binary aqueous methanol (MeOH), EtOH, acetone, TFE, and TFE-EtOH mixtures, the rate ratio oscillates within the very small range of 0.435–0.810. On the other hand in the aqueous HFIP mixtures, the trend is completely changed and the magnitude of the range of the rate ratio (*k***_1_**/*k***_4_**) increases exponentially from 0.417 in 70% HFIP to 356 in 97% HFIP. Furthermore, an inspection of the kinetic data reveals that *k***_4_** > *k***_1_** in MeOH, EtOH, acetone, TFE, TFE-EtOH, and 70% HFIP, and *k***_1_** >> *k***_4_** in 97% HFIP and 90% HFIP. This examination of the kinetic rates advances an interpretation that the inductive effect of the propargyl group in **4** is dominant in MeOH, EtOH, acetone, TFE, TFE-EtOH, and 70% HFIP, and that **1** and **4** must solvolyze by a similar mechanism with solvent nucleophilicity playing a significant role in these solvents. On the other hand in 97% HFIP and 90% HFIP, both highly ionizing solvents, the electron donating effect of the γ-methyl group in **1** predominates and the mechanism has now changed over to one where there is significant ionization in the transition state which is stabilized due to the presence of the electron supplying γ-methyl group.

In Table 2, we list the specific rates of solvolysis obtained for 2-methoxyphenyl chloroformate (**2**) in 17 solvents with broadly differing nucleophilicity and ionizing power values. Also recorded in Table 2, are the previously published pseudo first-order rate data for phenyl chloroformate (**3**) [50,51,53,54], 4-methoxyphenyl chloroformate (**5**) [48,49,53–55], and *p*-nitrophenyl chloroformate (**6**) [24,52,53].

An inspection of the kinetic data for **2** displays a tendency of the rates of solvolysis to increase with the addition of water to the aqueous organic mixtures, or with an increase in ethanol content in the TFE-EtOH mixtures. This observation also steers us towards a mechanism where solvent nucleophilicity plays a greater role in the rate determining step.

A comparison of the specific rates of solvolysis of **2**, **3**, **5**, and **6**, shown in Table 2, exposes a rate trend where *k***_6_** >> *k***_3_** > *k***_5_** > *k***_2_** in the aqueous methanol, ethanol, and acetone solvents. This rate direction suggests that the inductive effect of the phenoxy group is further greatly enhanced by the presence of a strongly deactivating nitro group in the *para* position in **6** making the carbonyl carbon highly susceptible to nucleophilic attack. PhOCOCl (**3**) is much faster than its methoxy-substituted analogs **2** and **5** in the more nucleophilic aqueous MeOH, EtOH, and acetone solvents, as the methoxy group can be electron-withdrawing inductively or electron-donating due to resonance. In these solvents, the rate data indicates that the methoxy group exhibits much greater electron withdrawing character in **2** and **5**, and is more efficient inductively in **5** due to a through-space (field) effect.

In the strongly hydrogen bonding solvent 97% TFE, the rate trends orient differently to *k***_6_** > *k***_2_** ≥ *k***_3_** > *k***_5_**. This suggests that in 97% TFE, the magnitude of the decrease in the inductive abilities of the *para* substituents in **6** and **5** is in all probability due to the presence of intermolecular hydrogen bonding between the solvent (TFE) and the oxygen atom(s) in the methoxy groups in **5** and in the nitro group in **6**.

In Table 3, we report the G-W analyses for substrates **1**–**6** using Equation 2 and employing the *N*_T_ [18–20] and *Y*_Cl_ scales [13–17]. For **1** in all the 18 solvents studied, we report an *l* value of 0.78 ± 0.18, a *m* value of 0.31 ± 0.12, and an intercept (*c*) of −0.15 ± 0.17. The correlation coefficient *R* = 0.832 and *F*-test = 17 is rather poor for such an analysis and is indicative of a superimposed mechanism occurring within the solvents. As mentioned earlier, trends in the rate data of **1** led us to conclude that this mechanistic change occurs in the highly ionizing HFIP mixtures. Removal of the four aqueous HFIP data points (*n* =14 solvents), leads to a *l* value of 1.50 ± 0.15, a *m* value of 0.49 ± 0.08, *c* = 0.15 ± 0.10, and very much improved *R* and *F*-test values of 0.956 and 58 respectively.

**Figure 2:**
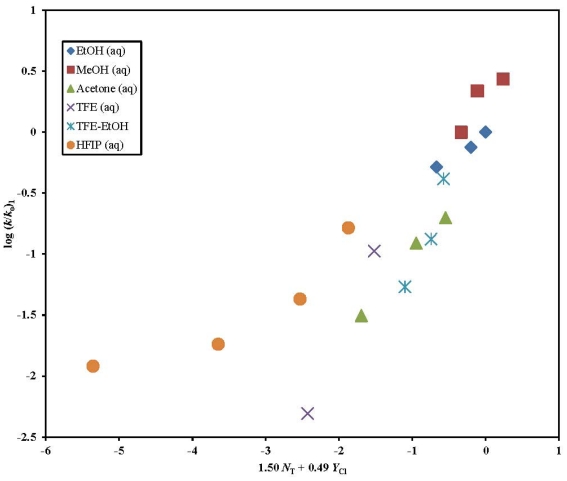
The plot of log (*k*/*k*_o_) for **1** against 1.50 *N*_T_ + 0.49 *Y*_Cl_.

A plot of log (*k*/*k*_o_)**_1_** against 1.50 *N*_T_ + 0.49 *Y*_Cl_ is shown in Figure 2. The four HFIP points (97–50% HFIP) were not included in the correlation but were added in the figure to show the extent of their deviation. In the remaining 14 solvents, the *l*/*m* ratio of 3.06 observed is very similar to the 2.96 reported for **3** in 49 solvents. This leads us to believe that **1** like **3** solvolyzes by an addition-elimination mechanism with a rate-determining addition step in the aqueous ethanol, methanol, acetone, TFE, and TFE-EtOH mixtures studied.

**Figure 3:**
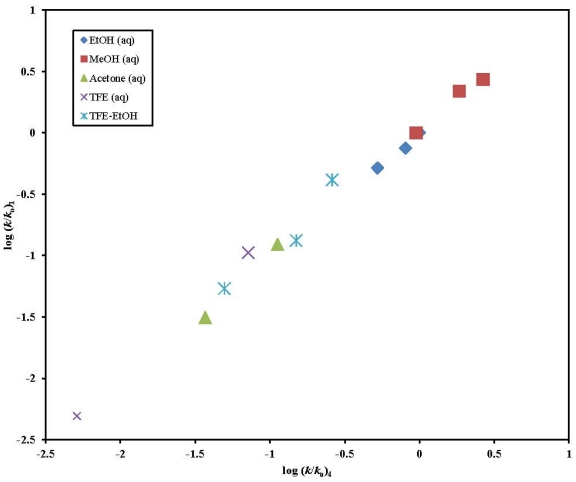
The plot of log (*k*/*k*_o_) **1** against the log (*k*/*k*_o_) values of **4** in the binary aqueous mixtures of EtOH, MeOH, acetone, TFE, and TFE-EtOH.

There are 13 common identical solvents (excluding the HFIP mixtures) in which substrates 2-butyn-1-yl chloroformate (**1**) and propargyl chloroformate (**4**) were investigated at 25.0 °C. For **1** and **4**, the similarity of mechanism in the thirteen identical solvents is confirmed by the good linear plot of log (*k*/*k*_o_)**_1_** against log (*k*/*k*_o_)**_4_** shown in Figure 3. The excellent linearity of the plot is affirmed by the goodness-of-fit parameters with a multiple correlation coefficient (*R*) of 0.995, an *F*-test value of 1075, a slope of 1.01± 0.03, and an intercept of 0.03 ± 0.03. The *l*/*m* ratio of 2.98 for **1** and 3.10 for **4** is strongly indicative of an analogous rate-determining formation of a tetrahedral intermediate in the 13 common solvents.

Aforementioned the observation that *k***_1_** >> *k***_4_** occurs in the 97% and 90% HFIP mixtures that exhibit strong hydrogen bonding, is characteristic of a unimolecular ionization transition state which materializes due to the presence of the electron-donating γ-methyl group in **1**. The second observation that the specific rates of reaction of **1** increase with the increase in water content in aqueous HFIP solvents is similar to the progressions seen for the ionization-type mechanisms witnessed in the HFIP mixtures in other alkyl [12,36,68–70] and alkenyl [37] chloroformate esters. This rate trend denotes that the developing unimoleular carbocation is stabilized by strong rear-side nucleophilic solvation.

For the 70% HFIP mixture, the *k***_1_**/*k***_4_** ratio falls to 0.42, a similar value to that observed for solvolyses in mixtures of water with methanol, ethanol, and acetone. This suggests a changeover from the ionization mechanism indicated in solvents richer in HFIP to the addition-elimination pathway.

Using the LFER relationship log (*k*/*k*_o_)**_1_** = 1.50 *N*_T_ + 0.49 *Y*_Cl_ + 0.15 (*l*, *m*, and *c* values from Table 3), we calculated the rate measurements for the HFIP mixtures that would be expected if **1** strictly followed the bimolecular addition-elimination pathway in these solvents. Our computations resulted in values of 2.12 × 10^−9^, 1.22 × 10^−7^, 1.41 × 10^−6^, and 6.46 × 10^−6^, for 97, 90, 70, and 50% HFIP respectively. From a comparison of these calculated values to those experimentally determined and reported in Table 1, one can calculate the corresponding % ionization values for **1** in 97, 90, 70, and 50% HFIP. These are determined to be 100%, 98%, 10%, and 12% respectively.

**Figure 4:**
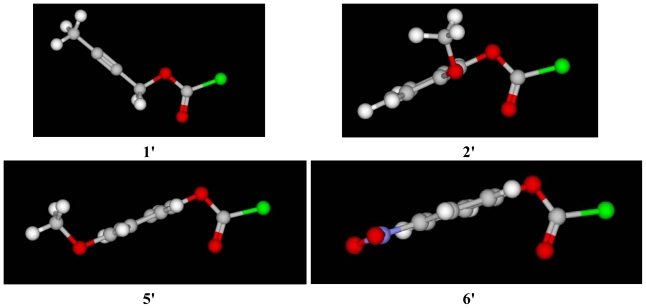
The 3-D images for the *syn* conformers of 2-butyn-1-yl chloroformate (**1′**), 2-methoxyphenyl chloroformate (**2′**), 4-methoxyphenyl chloroformate (**5′**), and 4-nitrophenyl chloroformate (**6′**).

As shown in the 3-D image **1′** for the *syn* conformer of **1** in Figure 4, the electron-supplying ability of the γ-methyl group on the β-alkyne in **1** is very much reduced since this alkynyl group is twisted out of the plane of the ester oxygen due to the presence of the methylene carbon. It is due to this orientation of the groups in space that the ionization pathway remains dominant only in the very strongly hydrogen bonding 97 and 90% HFIP mixtures.

**Figure 5:**
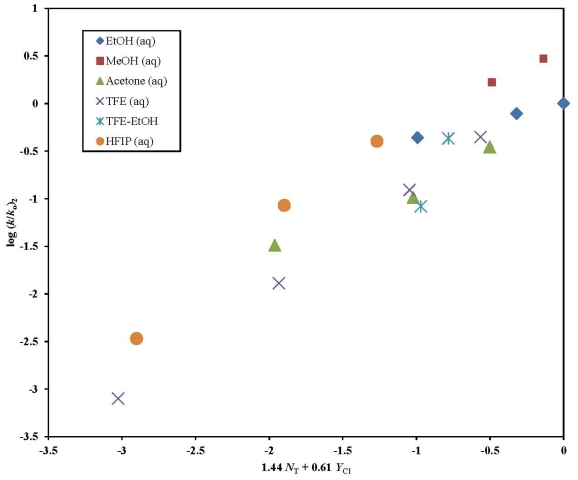
The plot of log (*k*/*k*_o_) for 2-methoxyphenyl chloroformate (**2**) against 1.44 *N*_T_ + 0.61 *Y*_Cl_.

For *o*-methoxyphenyl chloroformate (**2**), the G-W analyses (Table 3) in the 17 solvents studied resulted in *l* = 1.44 ± 0.16, *m* = 0.61 ± 0.10, *c* = 0.32 ± 0.14, *R* = 0.941, and *F*-test = 54. The slightly inferior correlation seen in the G-W analyses of **2** can be explained by the dual competing mesomeric and inductive effects operating simultaneously at the *ortho* position of **2**. A plot of log (*k*/*k*_o_)**_2_** against 1.44 *N*_T_ + 0.61 *Y*_Cl_ that is shown in Figure 5 does show a slight scatter with the highly ionizing aqueous HFIP mixtures lying slightly above the regression line. Removal of the three HFIP points results in *l* = 1.49 ± 0.12, *m* = 0.55 ± 0.07, an *l*/*m* ratio of 2.71, *c* = 0.30 ± 0.10, *R* = 0.972, and an improved *F*-test value of 93 in the remaining 14 solvents.

For all of the 17 solvents that **2** was studied in, the observed *l*/*m* ratio of 2.36 falls within the range that is typically observed in the solvolyses of chloroformate esters [12,21,22–26,28–39,41,42,50,54, 55,68–70] where the addition-elimination pathway with rate determining addition predominates. We also list in Table 3 the previously published *l*/*m* ratios of 2.96 for phenyl chloroformate (**3**) [50,54], 2.81 for *p*-methoxyphenyl chloroformate (**5**) [55], and 3.65 for *p*-nitrophenyl chloroformate (**6**) [24], in 49, 44, and 39 solvents respectively.

The *l*/*m* ratios of **3** and **5** in the identical 17 solvents that **2** was analyzed in, are, 2.93 and 2.81 respectively (Table 3). The gradual receding seen in the *l*/*m* ratio going from 2.93 in **3**, to 2.81 in **5**, and then to 2.36 in **2**, is befitting of the decrease in inductive ability and a decrease in the importance of general base catalysis in going from **3** to **5** to **2**.

Also reported in Table 2 are the rate ratios *k***_5_**/*k***_2_** in the 17 common solvents. In methanol, ethanol, and acetone, these ratios range from 2.91 at the low end to 6.07 in the polar aprotic 90% acetone. As explained earlier, such differences in rate ratios are consistent with the earlier suggestion that the methoxy group in substrate **5** exhibits an increased inductive capacity. Also in these nucleophilic solvents steric effects could account for the fact that **2** is slower than **5**.

This expansion in the electron-withdrawing character observed when the methoxy group is in the *para* position in **5** is acutely transmitted by the 3-D views of 2-methoxyphenyl chloroformate (**2′**) and 4-methoxyphenyl chloroformate (**5′**) shown in Figure 4. In the conformer **5′**, the electron-cloud distribution between the methoxy and the ester oxygens is completely planar and as a result the carbonyl carbon becomes much more electron deficient. This inductive ability of the substituted phenoxy group is further magnified in *p*-nitrophenyl chloroformate (**6**) due to the coplanarity of the *para*-nitro substituent and the ester oxygen, as shown in its conformer **6′**.

On the other hand in the strong hydrogen bonding solvents, especially in 90, 97% TFE, and 90% HFIP, the rate trend changes to *k***_2_** becoming slightly greater than *k***_5_**. This is due to the decrease in the inductive ability of the *p*-methoxy group in **5** because of the increased intermolecular hydrogen bond formation between the acidic hydrogen of the solvent group and the oxygen atom of this *p*-methoxy group.

**Figure 6:**
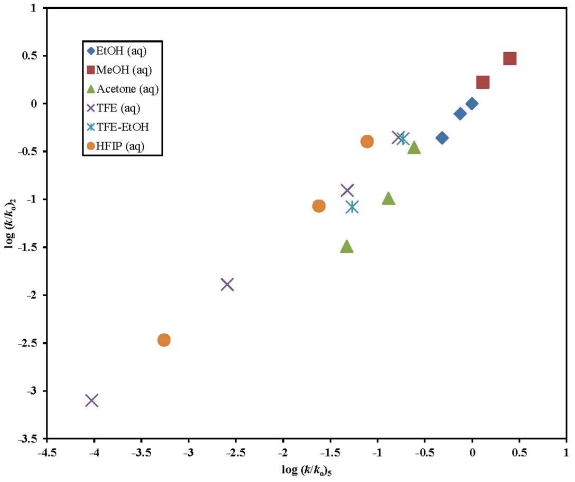
The plot of log (*k*/*k*_o_) for 2-methoxyphenyl chloroformate (**2**) against 4-methoxyphenyl chloroformate (**5**).

The plot of log (*k*/*k*_o_)**_2_** versus log (*k*/*k*_o_)**_5_** is shown in Figure 6. In the 17 solvents, there is a good correlation coefficient (*R*) of 0.975, *F*-test of 288, the slope is 0.778 ± 0.05, and the intercept is 0.05 ± 0.07. This satisfactory linear correlation further ascertains that **2** and **5** solvolyze by very similar mechanisms in all the solvents evaluated with a slightly earlier transition state for **2**.

## 3. Experimental Section

The 2-butyn-1-yl chloroformate (98%) and the 2-methoxyphenyl chloroformate (98%) were obtained from the Sigma-Aldrich Chemical Company and were used as received. Solvents were purified and the kinetic runs carried out as described previously [12]. A substrate concentration of approximately 0.005 M in a variety of solvents was employed. For some of the runs, calculation of the specific rates of solvolysis (first-order rate coefficients) was carried out by a process in which the conventional Guggenheim treatment [71] was modified [72] so as to give an estimate of the infinity titer, which was then used to calculate for each run a series of integrated rate coefficients. The specific rates and associated standard deviations, as presented in Tables 1 and 2, are obtained by averaging all of the values from, at least, duplicate runs.

Multiple regression analyses were carried out using standard Microsoft statistical packages [73] and calculations for the Guggenheim treatments were performed on commercially available software [74]. The 3-D images presented in Figure 4, were computed using the KnowItAll^®^ Informatics System [75].

## 4. Conclusions

Linear free energy relationships such as the G-W equations (Equations 1–4) are simple and useful teaching and practical tools in physical organic chemistry that can be used to interpret and elucidate the solvolytic mechanism of reaction efficiently. In this study, it is demonstrated that 2-butyn-1-yl chloroformate (**1**) solvolyzes by dual reaction channels in some of the solvents studied. In 14 solvents the addition-elimination dominates and, in the 2 strongly hydrogen bonding HFIP mixtures (97 and 90% HFIP), the reaction switches over to an ionization channel where the presence of the electron-supplying γ-methyl group stabilizes the developing carbocation.

The *k***_1_**/*k***_4_** ratios of 356 in 97% HFIP and 15 in 90% HFIP fall to a value of 0.42 in 70% HFIP, becoming essentially identical to values in aqueous methanol, ethanol, and acetone and consistent with a switch to an addition-elimination (association-dissociation) mechanism.

In 2-methoxyphenyl chloroformate (**2**) the inductive and mesomeric effects of the methoxy group compete simultaneously resulting in a slightly inferior G-W plot. The *l*/*m* ratio of 2.36 is within the range that would be expected for an aryl chloroformate ester that solvolyzes via a rate-determining addition step in a bimolecular addition-elimination process.

## Figures and Tables

**Table 1:** Specific rates of solvolysis (*k*) of **1** and **4** in several binary solvents at 25.0 °C and literature values for (*N_T_*) and (*Y_Cl_*).

Solvent (%) a	1 @ 25.0 °C; 10^5^*k*, s^−1^^b^	4 @ 25.0 °C; 10^5^*k*, s^−1^^b^,^c^	*k*_1_/*k*_4_	*N_T_*^d^	*Y_Cl_*^e^
100% MeOH	34.0 ± 1.2	63.4 ± 1.2	0.536	0.17	−1.2
90% MeOH	74.2 ± 2.5	123 ± 3	0.603	−0.01	−0.20
80% MeOH	92.9 ± 3.7	178 ± 10	0.522	−0.06	0.67
100% EtOH	17.6 ± 0.9	35.0 ± 0.8	0.503	0.37	−2.50
90% EtOH	25.6 ± 1.9	53.9 ± 1.2	0.474	0.16	−0.90
80% EtOH	34.2 ± 1.6	66.7 ± 1.6	0.513	0.00	0.00
90% Acetone	1.07 ± 0.04	2.46 ± 0.10	0.435	−0.35	−2.39
80% Acetone	4.21 ± 0.20	7.52 ± 0.22	0.560	−0.37	−0.80
70 % Acetone	6.77 ± 0.24			−0.42	0.17
90% TFE (w/w)	0.168 ± 0.020	0.342 ± 0.007	0.491	−2.55	2.85
70% TFE (w/w)	3.62 ± 0.17	4.78 ± 0.07	0.757	−1.98	2.96
60T-40E	1.84 ± 0.12	3.31 ± 0.01	0.556	−0.94	0.63
40T-60E	4.52 ± 0.21	10.0 ± 0.2	0.452	−0.34	−0.48
20T-80E	14.1 ± 1.4	17.4 ± 1.0	0.810	0.08	−1.42
97%HFIP (w/w)	0.413 ± 0.027	0.00116 ± 0.00009	356	−5.26	5.17
90%HFIP (w/w)	0.625 ± 0.029	0.0426 ± 0.0020	14.7	−3.84	4.41
70%HFIP (w/w)	1.47 ± 0.13	3.52 ± 0.18 f	0.417	−2.94	3.83
50%HFIP (w/w)	5.61 ± 0.25			−2.49	3.80

a Substrate concentration of *ca.* 0.0052 M; binary solvents on a volume-volume basis at 25.0 °C, except for TFE-H_2_O and HFIP-H_2_O solvents which are on a weight-weight basis. T-E are TFE-ethanol mixtures;

b With associated standard deviation;

c Reference [35];

d References [18–20];

e References [13–17];

f Listed erroneously in Table 1 of Reference [35]; the correct value (as here listed) was, however, used in the correlations.

**Table 2:** *k***_2_**, *k***_3_**, *k***_5_**, and *k***_6_**, in several binary solvents at 25.0 °C.

Solvent (%)^a^	2 @ 25.0 °C; 10^5^*k*, s^−1^^b^	3 @ 25.0 °C; 10^5^*k,* s^−1^^b^,^c^	5 @ 25.0 °C; 10^5^*k,* s^−1^^b^,^d^	*k*_5_/*k*_2_	6 @ 25.0 °C; 10^5^*k*, s^−1^^b^,^e^
100% MeOH	127 ± 17	695 ± 9	414 g	3.25	13500 h
90% MeOH	226 ± 14	1290 f	800 g	3.54	22700 h
100% EtOH	33.3 ± 2.7	260 ± 3	153 ± 4	4.59	5570 h
90% EtOH	59.7 ± 2.4	389 ± 6	239 g	4.00	11800 h
80% EtOH	76.3 ± 1.9	503 ± 11	318 g	4.17	13900 h
90% Acetone	2.47 ± 0.13	23.8 ± 1.4	15.0 ± 0.6	6.07	
80% Acetone	7.83 ± 0.17	68.8 ± 0.8	41.5 g	5.30	2050 h
70 % Acetone	26.6 ± 0.82	125 f	77.4 g	2.91	3190 h
97% TFE (w/w)	0.0605 ± 0.0021	0.0570 ± 0.0030	0.0300 ± 0.0013	0.492	0.113 ± 0.008
90% TFE (w/w)	0.976 ± 0.019	1.15 ± 0.08	0.825 ± 0.032	0.845	8.87 ± 0.28
70% TFE (w/w)	9.43 ± 0.24	17.4 ± 1.3	15.2 ± 0.6	1.61	153 ± 1.5
50% TFE (w/w)	33.7 ± 1.1	63.5 ± 3.0	52.6 ± 2.8	1.56	438 ± 44
60T-40E	6.39 ± 0.22	19.9 ± 0.5	17.0 ± 0.5	2.66	
40T-60E	32.7 ± 1.2	57.7 ± 1.9	59.2 ± 2.3	1.81	
90%HFIP (w/w)	0.258 ± 0.018	0.166 ± 0.004	0.175 ± 0.007	0.678	1.20 ± 0.06
70%HFIP (w/w)	6.48 ± 0.20	10.5 ± 0.3	7.58 ± 0.22	1.17	83.8 ± 0.9
50%HFIP (w/w)	30.5 ± 0.18	31.6 ± 0.6	24.9 ± 0.5	0.816	277 ± 2

a Substrate concentration of *ca.* 0.0052 M; binary solvents on a volume-volume basis at 25.0 °C, except for TFE-H_2_O and HFIP-H_2_O solvents which are on a weight-weight basis. T-E are TFE-ethanol mixtures;

b With associated standard deviation;

c References [50,54];

d References [54,55];

e Reference [24];

f References [51,53];

g References [48,49,53];

h References [52,53].

**Table 3:** Correlation of the specific rates of reaction of **1–6** using Equation 2.

Substrate	*n*^a^	*l*^b^	*m*^b^	*l/m*	*c*^c^	*R*^d^	*F*^e^
**1**	18	0.78 ± 0.18	0.31 ± 0.12	2.52	−0.15 ± 0.17	0.832	17
	14 f	1.50 ± 0.15	0.49 ± 0.08	3.06	0.15 ± 0.10	0.956	58
	13 f,g	1.52 ± 0.14	0.51 ± 0.07	2.98	0.18 ± 0.10	0.963	63
**2**	17	1.44 ± 0.16	0.61 ± 0.10	2.36	0.32 ± 0.14	0.941	54
**3**	49 h	1.66 ± 0.05	0.56 ± 0.03	2.96	0.15 ± 0.07	0.980	568
	17 i	1.58 ± 0.17	0.54 ± 0.10	2.93	0.21 ± 0.15	0.958	79
**4**	22	1.37 ± 0.10	0.47 ± 0.07	2.91	0.11± 0.11	0.970	152
	13 f,g	1.49 ± 0.13	0.48± 0.07	3.10	0.14 ± 0.09	0.969	77
**5**	44 j	1.60 ± 0.05	0.57 ± 0.05	2.81	0.18 ± 0.06	0.981	517
	17 i	1.60 ± 0.17	0.57 ± 0.10	2.81	0.26 ± 0.15	0.958	78
**6**	39 k	1.68 ± 0.06	0.46 ± 0.04	3.65	0.074 ± 0.08	0.976	363

a *n* is the number of solvents;

b With associated standard error;

c Accompanied by standard error of the estimate;

d Correlation coefficient;

e *F*-test value;

f No HFIP (aq) mixtures;

g To compare with **1** and **4** in common identical solvents;

h References [50,54];

i To compare with **2** in common identical solvents;

j Reference [55];

k Reference [24].
